# Alkaloids as potential antivirals. A comprehensive review

**DOI:** 10.1007/s13659-022-00366-9

**Published:** 2023-01-04

**Authors:** Shah Faisal, Syed Lal Badshah, Bibi Kubra, Abdul-Hamid Emwas, Mariusz Jaremko

**Affiliations:** 1 Department of Chemistry, Islamia College University Peshawar, Peshawar, 25120 Pakistan; 2 Core Labs, King Abdullah University of Science and Technology (KAUST), Thuwal, 23955-6900 Saudi Arabia; 3 Division of Biological and Environmental Sciences and Engineering (BESE), Smart-Health Initiative (SHI) and Red Sea Research Center (RSRC), King Abdullah University of Science and Technology (KAUST), Thuwal, 23955-6900 Saudi Arabia

**Keywords:** Alkaloid antivirals, Antiviral agents, Antiviral phytochemicals, In vitro, Spread, Inhibition

## Abstract

Alkaloids are a diverse group of natural phytochemicals. These phytochemicals in plants provide them protection against pests, and herbivorous organisms and also control their development. Numerous of these alkaloids have a variety of biological effects, and some have even been developed into medications with different medicinal properties. This review aims to provide a broad overview of the numerous naturally occurring alkaloids (isolated from both terrestrial and aquatic species) along with synthetically produced alkaloid compounds having prominent antiviral properties. Previous reviews on this subject have focused on the biological actions of both natural and synthetic alkaloids, but they have not gone into comprehensive detail about their antiviral properties. We reviewed here several antiviral alkaloids that have been described in the literature in different investigational environments i.e. (in-vivo, in-ovo, in-vitro, and in-silico), and found that these alkaloid compounds have significant antiviral properties against several infectious viruses. These alkaloids repressed and targeted various important stages of viral infection at non-toxic doses while some of the alkaloids reported here also exhibited comparable inhibitory activities to commercially used drugs. Overall, these anti-viral effects of alkaloids point to a high degree of specificity, implying that they could serve as effective and safe antiviral medicines if further pursued in medicinal and pharmacological investigations.

## Introduction

Alkaloids are a broad group of naturally occurring organic compounds, forming most of the largest group of phytochemicals [1]. Nitrogen atoms are a key distinguishing trait of alkaloids resulting in their alkaline properties and medicinal actions [2]. Amino acids such as tyrosine, lysine, ornithine, phenylalanine, and tryptophan are used to synthesize most alkaloids. These precursors are transformed into a variety of core intermediates, which lead to the production of numerous alkaloids. Alkaloids come in many different chemical forms, the majority of which have heterocyclic tertiary nitrogen in their structure. There are around 20,000 known alkaloids, mostly derived from plants. Microorganisms, marine organisms including algae, puffer fish, dinoflagellates, and terrestrial animals like insects and toads have all also been shown to contain alkaloids [3].

Plant species that possess above 0.001% alkaloids are referred to as alkaloids sources. As a result, plant groups such as *Solanaceae*, *Fabaceae*, *Asteraceae*, *Papaveraceae*, *Amaryllidaceae*, *Rutaceae*, *Apocynaceae*, and *Rubiaceae* have the potential to be utilized in pharmaceuticals [4]. The two major classes of alkaloids, indole alkaloids and isoquinoline alkaloids (each with about 4000 molecules) are generally categorised according to their chemical skeleton. Pyrrolizidine, tropane, pyridine, and steroidal alkaloids are other prominent classes of alkaloid phytochemicals. Alkaloids can also be classified by their botanical source. For example, papaver plants belong to the *Papaveraceae* family and contain (opium) alkaloids. Cinchona plants belong to the *Rubiaceae* family, famous for cinchona alkaloids. Other botanical sources include alkaloids from Rauvolfia, Catharanthus, Strychnos, Ergot, and cactus plants [3]. Alkaloids such as theobromine and caffeine, found in coffee, cacao seeds, and tea leaves, are consumed worldwide by humans daily [3]. In humans, many alkaloids have potent biological effects. This can be explained in part by the structural similarities between dopamine, noradrenaline, serotonin, and acetylcholine, which are all key neuro-transmitters. Alkaloids have unique features for medical usage since they are water-soluble in acidic conditions and fat-soluble in neutral and alkaline conditions.

In contrast to their edible use, alkaloids have a long history in human medicine and are often used to treat neurological problems [5], cancer [6], metabolic disorders [7], and infectious diseases [8]. Alkaloid phytochemicals may also help with antiviral treatment. Existing antiviral medications, on the other hand, have limited antiviral activity and variable toxicity toward patients, limiting their effectiveness. Phytochemicals such as alkaloids have a variety of biological and physiological functions and can be utilised as medications on their own [9]. Another possibility is that we can synthesize new drugs based on natural alkaloids.

Attempts in the past have been made various times for reviewing the antiviral mode of alkaloid compounds [10–13] but these past reviews either focus on the activity of alkaloids against a single virus or on the activity of a single antiviral alkaloid against different viruses, while some reviews include a mix of different phytochemicals along with alkaloid compounds and some of the reviews contains significantly good information about antiviral alkaloids but the set of reviewed alkaloid compounds and the viruses discussed/analyzed are too numerous and fewer.

In this review, we will be specifically and only discussing alkaloids that only act on pathogenic viruses and will focus on comprehensively reporting the different modes and mechanisms of actions along with their inhibitory concentrations, and will also be reporting the experimental models/Environments like in-vivo, in-vitro, and in-ovo which were utilized in studying these antiviral alkaloids against these pathogenic viruses.

The information analyzed in this review is collected by searching different research search engines like Google Scholar, and Symantec scholar along with other publishers linked research databases like Science direct, ACS journals, Wiley, Springer-Link linked research databases and Web of Science databases while independent research database and social research networks like Research Gate is also utilized along with these previously mentioned literature resources. Research articles, Review articles, and Patents mentioning the antiviral mode of alkaloids from 1960 till mid 2022 were accessed from all of these sources and then subjected to further analysis.

The analysis of all these literature materials resulted in shortlisting over a hundred different alkaloids of both natural and synthetic nature which were active against Twenty-Eight (28) different pathogenic viruses. Out of these reported and analyzed alkaloids compounds 90 compounds showed good activities against the target viruses and about eleven alkaloids that act on different viruses out of these total ninety alkaloids are found to be of synthetic nature while all the other alkaloids were of natural origin.

### Antiviral properties of alkaloids

**Fig. 1:**
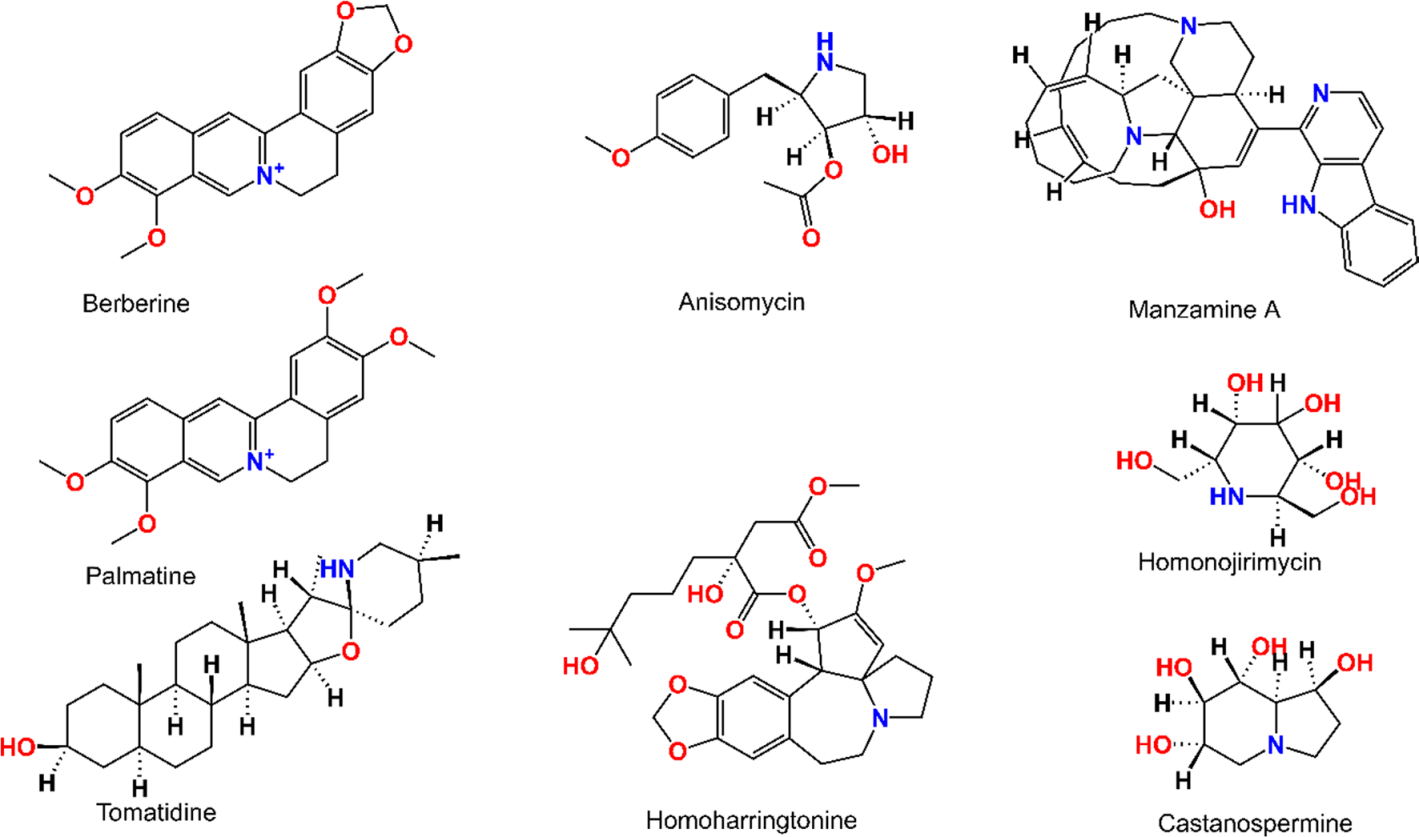
Structures of some antiviral alkaloids

Viruses carry DNA and RNA as their genetic material, as well as protein envelopes in some cases. To multiply and thrive, they rely on the metabolism of their hosts and their environment. They exploit and take over the host’s cellular machinery, spreading throughout the organism [14]. Alkaloid phytochemicals can suppress and act on viruses through a variety of methods. They can impede viral fusion or attachment with the host cell surface receptors preventing entry into host cells. They can also interfere with viral DNA or RNA synthesis [15], protein synthesis, and viral protein assembly [16]. They can also prevent viruses from infecting other healthy cells in the host by targeting transport mechanisms. The chemical structures of some of the antiviral alkaloids are given in Fig. 1.

In this review, the antiviral role of some alkaloids of different origins against a variety of viruses is presented.

## Antiviral activities of alkaloids against multiple viruses

### Alkaloids against influenza virus (IAV)

Influenza A viruses are single-stranded, negative-sense RNA viruses in the Orthomyxoviridae family. Seasonal influenza A strains generate major morbidity and economic losses around the world each year. In most cases, influenza A (IAV) infects tracheal cells and as well the bronchial epithelial cells, causing localised cellular damage and inducing an acute inflammatory response within the host [17].

Berberine alkaloid was studied in vitro for their effect on IAV infections. Berberine can suppress IAV type A/PR/8/34 in the RAW-264.7 cells at over 1 μM, while the IC_50_ for berberine was 0.01 μM. This alkaloid also suppressed the growth of a separate strain of H1N1 IAV in vitro, with an IC_50_ of 0.44 μM. Berberine’s mode of action implies that it affects virus protein maturation as well as its transportation, which in turn slows virus development. Berberine was also found to inhibit the production of tumour necrosis factor-a (TNF-a) and prostaglandin E2 (PGE2) in H1N1 (PR8) infected cells [18]. Berberine was also studied by other researchers both in vitro and in a mouse model for its antiviral properties. In this study, berberine attenuated the cytopathic effect (CPE) of IAV in infected MDCK cells and lowered viral protein neuraminidase (NA) activity with an IC_50_ of 0.025 g/L. Berberine significantly reduced mortality, increased mean survival time, and decreased viral titers in IAV-infected mice. Berberine significantly reduced the degenerative alterations in the lungs of mice and showed a direct repressive effect on IAV infection in vitro and in vivo [19].

Another research team isolated Homonojirimycin (HNJ), an alkaloid from the *Commelina communis* L., and tested its antiviral efficacy on the IAV/PR/8/34 (H1N1) strain. HNJ had a substantial antiviral efficacy against IAV, with an inhibitory concentration EC_50_ of 10.4 μg/mL [20]. This same alkaloid was again tested for its efficacy in the in vivo mice model for its effect on the IAV; HNJ managed to enhance the survival rate, prolong the mean survival time, and lower virus production in the lungs of mice on days 4 and 6 post-infection, as per the findings of this study, after the compound was orally delivered to the mice from 2 days prior inoculation to 6 days after infection. On days 2, 4, and 6, mice infected with IAV had significantly higher levels of interferon (IFN) and interleukin (IL)-10 in their serum and lungs, but lower levels of TNF and IL-6. These findings demonstrated that HNJ protected the mice against IAV infection and elicited efficient immune responses in the In vivo studies [21].

Alkaloids derived from marine micro-organisms were also reported to have notable antiviral effects on the IAV. Oxoglyantrypine alkaloid derived from the marine mangrove plant-fungal strain *Cladosporium* sp. was noted to have a repressive effect against the IAV with an IC_50_ of 85 μM; other alkaloids derived from this fungal strain were Norquinadoline A, Deoxynortryptoquivaline, Deoxytryptoquivaline, Tryptoquivaline had also a reported inhibitory effect with an IC_50_ of 82, 87, 85, 89 μM, respectively, against IAV [22].

Synthetic alkaloids prepared by a single-stage synthesis procedure based on the quinazoline alkaloid are also reported to have potent antiviral activity against IAV. An alkaloid prepared from the (+)-camphoric acid which is a Quinazoline alkaloid’s synthetic analogue, had significant antiviral activity against the IAV A/Puerto Rico/8/34 (H1N1) strain with an IC_50_ of 17.9 ± 2.0 μM. Its CC_50_ concentration was > 1117.9 μM towards the cells in which the virus was inoculated. Further, this compound also showed stronger inhibition of other IAV strains “A/Aichi/2/68 (H3N2) and A/mallard/Pennsylvania (H5N2)” with IC_50_ values of 27 ± 4 and 21 ± 3 μM respectively [23].

### Alkaloids against herpes simplex virus-I and II (HSV-I and II)

HSV is classified into two serovars: HSV-I and II. HSV-I infections are usually accompanied by moderate to severe symptoms such as blisters and swelling of cells in the mouth and eyes, and in certain situations can cause more serious conditions such as blindness, hearing loss, and fatal encephalitis. HSV-2 infections, on the other hand, can cause minor genital sores while significantly increasing the chance of contracting and transferring (HIV) and other opportunistic infections [24].

Several alkaloids have been reported to have a potent antiviral effect on HSV-I. In a study understanding the effects of different phytochemicals i.e. flavonoids, plant phenolics, and alkaloids, it was shown that alkaloids of several types show good antiviral activity against HSV-I in the MDBK cell line. Alkaloids tested in this experiment showed significant inhibition and reduction of the cytopathogenic effect (CPE) caused by this virus in MDBK cell culture with an inhibitory concentration of 0.8 µg/mL for Yohimbine, 0.8 µg/mL for vincamine, 0.8 µg/mL for atropine, 0.4 µg/mL for trigonelline and 0.4 µg/mL for capsaicin. These alkaloids had a maximum non-toxic concentration (MNTC) of < 3.2 µg/mL towards the MDBK culture cells. Other alkaloids used in this study also had a good repressive effect on the reduction of the CPE caused by HSV-I; scopolamine, allantoin, octopamine, synephrine, colchicine, and trigonelline alkaloids at a concentration of 1.6 µg/mL showed significant inhibitory activity on HSV-I [25].

Manzamine-A, another alkaloid, was tested for its repressive effect on the HSV-1 EGFP virus in rabbit corneal cells (SIRC). It was found that Manzamine-A exhibited an effective inhibitory activity on the HSV-I replication process at a 1 μM concentration in rabbit corneal cells (SIRC). The current anti-HSV-I medication acyclovir was also tested, and it exhibited similar inhibitory activity at 50 µM concentration. Moreover, Manzamine-A diminished the discharge of infectious viruses by a factor of 10^11^ in plaque assays. Manzamine A treatment reduced HSV-1 virion host shutoff function and also its ICP0 transcription process, according to RT-PCR tests. The IC_50_ of Manzamine-A was reported to be 5.6 µM against this virus. These findings point to manzamines as a promising lead for reducing viral infection in corneal cells and preventing eye infections including keratitis induced by HSV-I [26].

A study in BALB/c mice assessed the inhibitory action of the Tetrandine alkaloid against HSV-I virus-induced keratitis (HSK). The mice were infected in the right cornea and administered via the intraperitoneal route with either Tetrandine or Acyclovir. 45% of the mice treated with Tetrandine developed HSK when treatment was started on day 0 of infection, while Acyclovir inhibited the HSK infection when its treatment was started at the same time. But when the Tetrandine treatment was started on day 7 of the infection it reduced the HSK infection in the mice to 8.5% compared to 45% when given on day 0. Further analysis revealed that Tetrandine had no direct antiviral effect on HSV-I and that this decrease in HSK infection incidence was due to immune system modulation, suppressed HSV-I antibody production, and a reduction in inflammatory responses. These down-regulatory actions of Tetrandine on the anti-herpes immune response imply that this alkaloid can be used to control an early exuberant inflammatory response without compromising virus clearance from the eye. This could be a significant step forward in the treatment of herpes ocular infections [27].

Another in vivo study involving 6-*O*-butanoyl castanospermine alkaloid used a zosteriform model in mice. Oral treatment with this alkaloid delayed the development of lesions caused by the SC-16 strain of HSV-I. Additionally, the amount of virus isolated from the brain of mice was also decreased. In comparison to untreated controls, the viral load inside the brain tissue of mice who received treatment 2 days before infection was reduced by a factor of 100. The IC_50_ of 6-*O*-butanoyl castanospermine against HSV-I was 15 ± 4.8 µM when given before infection and 37 ± 5.5 µM when given after [28].

The alkaloid homoharringtonine (HHT) has also been reported to repress the HSV-I viral replication in Vero cells at a concentration ranging from 500 to 1000 nM. This alkaloid induced drops of one to three orders of magnitude on the infection of the HSV-1. When the mechanism of action of HHT was investigated, it was discovered that ICP8, a single-strand DNA binding protein (SSB) of HSV-I that is required for viral replication, was targeted, and upon analysis, a significant reduction of this protein was detected. On HSV-1 infection, the IC_50_ concentrations of HHT and acyclovir noted were 139 nM and 789 nM, respectively. HHT also reduced the level of eLF4E-protein (eIF4E is a cap-binding protein that attaches to mRNA in conjunction with other proteins like the helicase eIF4A and the scaffolding protein eIF4G, facilitating ribosome recruitment and for the commencement of protein translation process) [29] indicating that HHT inhibits HSV-1 by lowering the degree of phosphorylation of endogenous and exogenous eIF4E [30].

Tubingensin A—an alkaloid isolated from *Aspergillus tubingensis* (R.Mosseray 1934)—was also reported to have activity against HSV-I in an in vitro experiment, with an IC_50_ of 8 µg/mL [31]. Berberine alkaloid isolated from *Coptidis rhizome* (a rhizome of *Coptis chinensis* Franch.) was used in an in vitro study on both the HSV-I and II viral strains in Vero cells. It reported that berberine targeted the synthesis of the HSV viral fusion protein which is essential for entry to the host cells during infection. These fusion proteins gB and gE are also known as HSV virus late gene products. The IC_50_ concentrations of berberine against HSV-I and II were 8.2 ± 1.2 × 10^–2^ and 8.2 ± 1.2 × 10^–2^ mg/mL respectively and the CC_50_ concentration of berberine was 13.2 ± 1.6 mg/mL for Vero cells [32].

β-carboline alkaloids (Harmine and Compound-4 as reported in the publication), isolated from the seeds of *Peganum harmala* L. family Zygophyllaceae showed strong repressive activities in an HSV-II plaque reduction assay. Harmine and Compound-4 effectively reduced the plaque-forming units of both HSV-I and II with IC_50_ concentrations of 4.06 ± 0.68 and 2.12 ± 0.14 μM respectively. The CC_50_ concentrations for Harmine and Compound-4 were 87.15 ± 0.79 and 74.17 ± 0.62 μM respectively [33].

### Alkaloids against dengue virus (DENV)

Dengue virus (DENV) is a flavivirus. *Aedes aegypti* and *Aedes albopictus* mosquitoes spread DENV when people are bitten by these mosquitoes resulting in dengue fever. DENV has an 11-kb positive-sense RNA-genome that encodes structural proteins such as capsid (C), envelope (E), and membrane precursor (prM), as well as several nonstructural proteins [34]. Dengue hemorrhagic fever and dengue shock syndrome are induced by four DENV serovars (DENV1-4), all of which are potentially deadly [35, 36].

Cherylline an alkaloid from *C. jagus*; J. Thomps (Liliaceae) extract has repressive activity on DENV. Cherylline had an EC_50_ of 8.8 μM against DENV_R2A_ replication in a renilla luciferase reporter assay. At the highest tested concentration, the cherylline CC_50_ was not reached at 250 μM. Moreover, to test cherylline inhibitory action against DENV_GFP_ (Dengue virus with a green fluorescent protein (GFP) gene), Huh7 cells were infected with DENV_GFP_ in the presence of increasing concentrations (from 0.6 to 100 μM) of cherylline. Cherylline was shown to be more effective than ribavirin at inhibiting DENV-induced infection, with an EC_50_ of 8 μM, analogous to its action on DENV_R2A_, compared to ribavirin’s EC_50_ of 100 μM. Further investigation on the mode of action of this alkaloid against wild-type DENV showed that DENV RNA levels were decreased [37].

The inhibitory activity of the alkaloid anisomycin was tested on DENV-2 in another research in multiple cell lines. Anisomycin exerted dose-dependent inhibition on DENV-2. The addition of anisomycin alkaloid to the infected cell culture showed 99.9% inhibition of DENV-2 when added at 8 h p.i, but when added later at 18 h p.i no inhibition was observed which indicates anisomycin exerts its inhibitory activity in the initial stages of the viral infection. Viral internalization was noted to remain unaffected, but the viral protein synthesis of DENV-2 was strongly inhibited by anisomycin. Immunofluorescence showed that anisomycin inhibited the expression of the viral E-glycoprotein of DENV-2. Furthermore, qRT-PCR analysis showed that anisomycin alkaloid had a substantial inhibitory effect on DENV2 RNA synthesis at an inhibitory concentration of 200 nM [38].

Another alkaloid, castanospermine (an indolizidine alkaloid), was reported to have strong repressive action against DENV. When tested in both Huh-7 and BHK-21 cells, castanospermine inhibited DENV-2 with an IC_50_ of 85.7 μM and 1 μM respectively. Castanospermine was also able to inhibit other DENV serotypes. It was noted that this alkaloid affected the DENV virus-like particle (VLP) by interfering with the VLP incorporation of the prM-E and C-protein of DENV and reduced the DENV particles by more than 95%. Castanospermine also showed promising antiviral activity against DENV infection in a mouse model [39]. Tomatidine alkaloids also have anti-DENV activity. Tomatadine is non-toxic in the Huh 7 cell line, with a CC_50_ of 80.2 μM. 10 μM tomatdine repressed DENV virus particle production by 2.02 logs after infection at MOI 1. All the serotypes of DENV-1,2,3 and DENV-4 were effectively inhibited by tomatadine with EC_50_ concentrations ranging from 0.82 to 4.87 μM. In a time of addition assays, it was found that tomatidine inhibited DENV transmissibility both when added before, during, and up to 12 h after infection. A significant reduction in the expression of the DENV E-Protein was observed when treated with tomatadine. These findings imply that tomatidine is rapidly absorbed after being added to cells and likely performs its antiviral action shortly after virus entry [40].

Furthermore, three indole alkaloids (3-oxo-voacangine, voacangine-7-hydroxyindolenine, and rupicoline) isolated from *T. cymosa* L. (Apocynaceae) showed significant repressive activity against different DENV strains. These indole alkaloids have demonstrated direct virucidal inhibitory action in several reported research studies, these compounds also didn’t show any toxic effects on the different cell lines used in these studies. Moreover, its main parent alkaloid compound vocangine along with rupicoline and voacangine-7-hydroxyindolenine in a combinatorial therapy approach showed 90% inhibition of DENV-2 (NG and 16681) viral strains [41].

### Alkaloids against Chikungunya virus (CHIKV)

CHIKV is an alphavirus. It is an enclosed virus with a positive-sense single-stranded RNA-genome and a genome size of 11.8 kb. *Aedes aegypti* and *Aedes albopictus* are the main vectors that spread the virus to humans. CHIKV outbreaks have affected over two million individuals worldwide and resulted in hundreds of deaths [42].

In a chemical screen of compounds against CHIKV, it was reported that berberine alkaloid has strong anti-CHIKV activity in BHK-21 cells without affecting the viability of this cell line. A CHIKV replicon having the *Renilla* luciferase gene was used in this experiment. It was found that berberine acted on various stages of CHIKV infection. Berberine treatment downregulated the CHIKV viral protein synthesis and repressed the genomic as well as the antigenomic viral RNA synthesis. It was also reported that this alkaloid act on the replication process of CHIKV infection. The EC_50_ concentration of berberine against CHIKV was 1.8 μM [43].

Harringtonine alkaloid is also reported to have strong repressive activity against the CHIKV-122508 and CHIKV-0708 viral strains in BHK-21 cells. Harringtonine alkaloid treatment exerted dose-dependent inhibition of both CHIKV strains. It was reported that the CHIKV-122508 strain is more susceptible to inhibition by harringtonine compared to the CHIKV-0708 strain. Harringtonine targeted the CHIKV RNA and protein synthesis process, and qRT-PCR revealed that harringtonine significantly reduced the −ve and +ve sense RNAs of both the CHIKV strains. Western blot also revealed that the nsP3 and E2 proteins of CHIKV were dose-dependently inhibited by this alkaloid. The EC_50_ concentration of harringtonine against CHIKV was 0.24 μM and it showed minimal cytotoxicity towards the tested cell line [16].

Tomatadine alkaloid was tested in multiple cell lines against different CHIKV viral strains, and in Huh7 cells, it effectively prevented infection of three different CHIKV genotypes. With an EC_50_ value of 1.3 μM and a SI of 120, tomatadine repressed and inhibited the CHIKV La Reunion strain. EC_50_ values of 3.8 μM and 2.0 μM were established for the African and Asian CHIKV genotypes. Following time-of-addition investigations, it was discovered that the antiviral action occurs after infection and is maintained after the drug is added until six hours after infection [44].

### Alkaloids against Ebola virus (EBOV)

The Ebola virus, commonly known as the Zaire Ebola virus, was responsible for the Ebola virus outbreak. Since 1976, there have been several EBOV breakouts. EBOV possesses a single negative-strand RNA genome with a genomic size of 18,898 nucleotides. It is a member of the filoviridae virus family [45, 46]. Several alkaloids have been described to have repressive activity on EBOV. Emetine alkaloid was tested in multiple cell lines against EBOV. A viral-like particle of the VLP viral entry assay showed that emetine can effectively block EBOV VLP entry into HeLa cells in a dose-dependent fashion with an IC_50_ concentration of 10.2 μM. Moreover, the researchers also confirmed the repressive activity of emetine alkaloid in another live virus assay in Vero E6 cells. The IC_50_ of emetine in this assay against EBOV was 16.9 nM. An in vivo study in mice with mouse-adapted Ebola virus was also performed by these researchers. In contrast to the untreated mice which all died of EBOV, four out of six mice survived in the group treated with emetine alkaloid, confirming its efficacy in both experimental models. Similarly, cephaeline alkaloid which is emetine’s analogue also showed similar activity in the in vivo studies with an inhibitory concentration of 5 mg/kg/day when administered via the i.p. route [47].

In another in silico computational study involving piperine alkaloid, it was reported that during the Molecular docking studies this alkaloid showed stronger affinities towards the NS3 protease-helicase, methyltransferase, and the VP35 interferon inhibitory domain of EBOV. The interactions of piperine were much stronger than Ribavirin confirming that it can be a better choice against the EBOV according to molecular docking scores of this alkaloid with the viral proteins of EBOV [48].

### Alkaloids against human cytomegalovirus (HCMV)

Cytomegalovirus is a member of the Herpesvirales genus of the Herpesviridae family of viruses and is a beta herpes virus. It has a ds-DNA genome with a size of roughly 230 kbp and is enveloped. HCMV is an opportunistic virus that infects a host which has a compromised immune system, including AIDS patients and children, causing a variety of ailments.

In a LOPAC library screen against HCMV, emetine alkaloid had good antiviral activity as an HCMV inhibitor. Studies in human foreskin fibroblasts showed that emetine can effectively inhibit HCMV virus cell entrance but before DNA synthesis, resulting in lower viral protein expression. When emetine was used with ganciclovir, it produced synergistic viral inhibition. Emetine was well tolerated, had a long half-life, was distributed preferentially to tissues over plasma, and successfully inhibited the mouse-adapted CMV. Emetine's ability to inhibit HCMV was dependent on RPS14 binding to MDM2, which disrupted virus-induced MDM2-p53 and MDM2-IE2 interactions. The EC_50_ for emetine against HCMV was 40 ± 1.72 nM, with a CC_50_ of 8 ± 0.56 μM [49].

Berberine Chloride alkaloid has also been reported to have a potent anti-HCMV activity which is comparable to standard ganciclovir GCV antiviral drug. The plaque assay showed that HCMV can be potently inhibited by berberine. This alkaloid exhibited repressive HCMV activity with an IC_50_ of 0.68 μM and SI index of 110 in MRC-5 cells, while GCV drug has an IC_50_ of 0.91 μM against HCMV. It was noted that both the alkaloid berberine and GCV suppressed HCMV proliferation to a similar extent. An infection center assay showed that berberine has no influence on the entry of HCMV to host cells but interfered somewhere after entry and before the start of the viral DNA replication stage [50].

### Alkaloids against Zika virus (ZIKV)

Zika virus (ZIKV) is a Flavivirus with a genome size of 10.7 kb. ZIKV is an enveloped virus with positive-sense ssRNA as genetic material. *Aedes aegypti* and *albopictus* mosquitoes are the carriers of this virus. In young newborns, it causes disorders like brain microcephaly. ZIKV outbreaks have been documented all over the world [51–57].

A study with *C. jagus* J. Thomps (Liliaceae) alkaloid extracts showed a significant antiviral effect on the Zika virus. Upon further studies on the alkaloids present in this plant, it was found that Cherylline was the most active alkaloid with repressive activity on ZIKV. An experiment on ZIKV_R2A_ which has the Renilla luciferase reporter gene showed that Cherylline at EC_50_ of 20.3 μM inhibited the ZIKV_R2A_ replication with a selectivity index of > 12.3. Furthermore, the wild-type Zika strain H/PF/2013 and Zika virus MR766 instead of luciferase reporter infectious systems were used to certify the repressive properties of cherylline on the Zika virus. Cherylline treatment potently reduced the viral titer of the pathogenic H/PF/2013 strain 100-fold and MR766 by 88 percent. Plaque-assay with wild-type viruses revealed that cherylline inhibits ZIKV life cycles efficiently. Moreover, the lycorine alkaloid used in this experiment also showed potent inhibition of the Zika virus with an EC_50_ concentration of 0.41 μM with an SI of 35.4 against this virus [37].

Anisomycin alkaloid was evaluated for its efficacy against ZIKV in multiple cell lines and an AG129 mouse model. Dose-dependent inhibition of ZIKV by anisomycin was observed. Anisomycin addition of up to 8 or 5 h p.i. caused a 99.99 percent reduction in Zika virus production, according to a time-course investigation. Its repressive effect decreased when added at 12 h p.i, indicating that this alkaloid acts on the initial and intermediate stages of viral infection of ZIKV. In the identification of target studies, it was revealed that the post-internalization step was influenced and not viral entry. Immunofluorescence showed that anisomycin potently targeted the viral protein synthesis of this virus with a 95.0 ± 3.0% drop in the number of fluorescent cells in Zika virus-infected cell cultures at 18 and 24 h p.i. The EC_50_ of this alkaloid against ZIKV (PRVABC59) was 7.9 ± 1.2 nM with an SI of > 11,900 in A549 cells and its CC_50_ was > 94000 nM. AG129 mice showed that anisomycin has a reverse dose inhibition effect on the virus; mice administered with 100 mg/kg/d of anisomycin showed earlier sickness signs, and more rapid mortality was seen in them, while 4 mg/kg/d of this alkaloid treatment delayed the signs of the diseases and also reduced the mortality rate of the mice [38].

Emetine alkaloid strongly repressed the Zika virus MR766 strain in HEK293 cells with an IC_50_ of 52.9 nM (confidence intervals of 35.4–73.2 nM). This emetine repressive effect is not ZIKV strain-specific as both PRVABC59 and the FSS13025 ZIKV strains are also inhibited. In immunofluorescence staining, emetine also inhibited the MR766 ZIKV strain along with the ZIKV PRVABC59 strain by targeting the synthesis of the E-protein in the SNB-19 cell line with an IC_50_ concentration of 29.8 nM and confidence intervals of 24.4 to 35.0 nM. Moreover, a ZIKA virus titer assay performed in Vero cells showed complete inhibition of the viral replication with an IC_50_ of 8.74 nM and confidence intervals of 7.4–10.7 nM. The CC_50_ of emetine for the HEK293 and SNB-19 cell lines was 180 nM and 86 nM respectively. The mechanism of action of emetine against ZIKV was the repression of the NS1 protein as well as NS5 (RdRp protein of ZIKV). Computational studies further predicted and showed with docking that emetine preferred attachment to an allosteric site near the priming loop of the N-pocket of Zika virus (RdRp), acting as a non-nucleoside inhibitor. Furthermore, emetine also showed promising anti-ZIKV activity in a mouse model. An analogue of emetine called cephaeline (an alkaloid) also showed antiviral activity against ZIKV with an IC_50_ = 52.9 nM [47].

Three alkaloids were obtained from the marine fungus *Fusarium* sp. L1, produced by supplementation of the fungal culture with L-tryptophan. In plaque assays, promising anti-ZIKV activities in the A549 cell line were noted for these alkaloids without affecting cell proliferation at the tested doses. Alkaloids fusaindoterpene-B alkaloid, JBIR-03, and 1,2-bis(1*H*-indol-3-yl)ethane-1,2-dione—all of which are indoloterpene alkaloids—showed strong repressive activity in the plaque reduction assay with EC_50_ concentrations of 7.5, 4.2, and 5.0 μM, correspondingly against the Zika virus [58].

Palmatine alkaloid tested against ZIKV in Vero cells which were infected with 400 pfu of Zika virus and then inoculated with varying concentrations of palmatine showed significant repressive activity. After 2 days of incubation of ZIKV with palmatine, qRT-PCR assays showed that palmatine doses ranging from 10 to 80 μM repressed and inhibited ZIKV RNA synthesis. A fluorescence focus assay also revealed that this alkaloid reduced ZIKV progeny production. Time of addition assays which are probably performed to identify the mechanism of actions of compounds against a target virus revealed that palmatine exerts significant inhibitory activity on ZIKV when added at the pre-infection, co-infection, and post-infection stages. Palmatine, therefore, acts on ZIKV binding, entry, and post-entry stages (viral replication) and also disrupts the stability of the Zika virus by binding with its envelope proteins. These results show that palmatine can be a promising multi-stage inhibitor of Zika virus infection [59].

### Alkaloids against SARS-CoV-2

Coronaviruses are also known as picornaviruses [60]. They carry a positive-sense RNA as genetic material and are commonly found in birds and other animals [61]. They cause a variety of disorders, the most prevalent of which are respiratory diseases including SARS-CoV-2 [62–65].

An in silico study involving molecular docking and MD simulation approaches identified several alkaloids which showed strong interactions with the SARS-CoV-2 Nsp-15 protein. Ajmalicine, aranotin, and piperine alkaloids were identified to be promising repressors of Nsp-15 of SARS-CoV-2. The alkaloids in this computational investigation could potentially be promising leads against this virus by targeting replication. Nsp-15 is an important protein of SARS-CoV-2 and interfering with these alkaloid-based compounds may impede viral replication [66].

An in vitro screening of alkaloid compounds (mostly antimalarial inhibitors) against SARS-CoV-2 showed that alkaloids and alkaloid-based compounds have substantial repressive activity. Chloroquine, hydroxy-chloroquine, and pyronaridine alkaloids-based drugs showed significant antiviral activities against SARS-CoV-2 having EC_50_ concentrations of 2.1 μM, 1.5 μM, and 1.8 μM respectively. Other compounds like desethylamodiaquine, mefloquine, and quinine also showed good repressive activities with EC_50_ values of 0.52 μM, 1.8 μM, and 10.7 μM. These results suggest that these compounds can be effective in inhibiting this rapidly mutating infectious virus [67].

Another bioinformatics study showed that camptothecin (CPT), a quinolone alkaloid found in *Camptotheca acuminata* Decne tree had good interactions with the two main enzymes M_PRO_ and RdRp of SARS-CoV-2. An in silico bioinformatics analysis revealed that CPT and its derivatives, toptecan and irinotecan, have a greater binding affinity towards Mpro and RdRp. Molecular docking studies also revealed that CPT engages the amino acids at K466 and K355 of the viral S-protein of SARS-CoV-2, compared to lopinavir, an antiviral drug that only made Van Der Waal’s interactions with the S-protein and also had no H-bond interactions. It can be concluded from this study that CPT can hinder and potentially block the association of the S-protein and the ACE-2 receptor and that CPT can be efficacious in controlling SARS-CoV-2 infection [68].

Other alkaloids have also been reported to have good binding affinities with important enzymes of SARS-CoV-2 in bioinformatics studies [69]. Schizanthine Z alkaloid was identified by in silico approaches to have a better binding affinity with the papain-like protease enzyme of SARS-CoV-2 compared to the Lopinavir antiviral drug [70]. Similarly, two other alkaloids—sophaline D and thalimonine have been identified to have good binding energies towards the main-protease (M_PRO_) enzyme, suggesting that these alkaloids can be potential inhibitors of this virus [71].

An in vitro study in which the efficacy of certain antiviral drugs and alkaloids was evaluated against SARS-CoV-2 in Vero E6 cells revealed that homorringtonine and emetine alkaloid can inhibit this virus with EC_50_ concentrations of 2.55 μM and 0.46 μM respectively. Moreover, it was also noted that when emetine alkaloid and remdesivir are administered together, they inhibited the viral yield by 64.9 percent at doses of 0.195 μM and 6.25 μM. This study shows that the use of alkaloids in combinational therapy may result in better clinical outcomes [72].

### Alkaloids against hepatitis B virus (HBV) and hepatitis C virus (HCV)

HBV is a major world health problem, and although a viable vaccine is available, it is still estimated that about 35 million people are continuously affected by this virus worldwide. HBV has a 3.2 kb relaxed circular-DNA genome. HCV affects about 3% of the world's populace and is the leading cause of chronic liver disease. HCV causes both acute and chronic hepatitis, with symptoms ranging from a brief illness to a serious, life-threatening condition [73, 74].

Alkaloids isolated from *Z. nitidum* (Roxb.) DC (Rutaceae) roots were evaluated for their anti-HBV activities in HepG2 2.2.15 cells The cultured cells were first transfected with HBV DNA to produce HBV viral particles. It was reported that the tested alkaloids had potent inhibitory activities against HBV. The extracted alkaloids 5,6-dihydro-6-methoxynitidine, skimmianine, and 5-methoxydictamine showed significant repressive anti-HBV effects by targeting the HBsAg and HBeAg secretions. The inhibitory concentrations were 0.2 μmol/mL for the first two alkaloids and this repressed the HBsAg secretions by 43.3 and 49.3 percent respectively. While skimmianine alkaloid also inhibited the HBeAg at 0.2 μmol/mL concentration, 5-methoxydictamine alkaloid targeted the HBeAg secretion and showed a 43.2% repressive effect against HBV. In contrast, the positive control lamivudine showed only 29.6 and 35.4 percent inhibitory activity at a concentration of 1.0 μmol/mL against the HBsAg and HBeAg secretions. These findings suggest that these alkaloids can be efficient in controlling HBV infection [75].

Similarly, alkaloids isolated from *Sophora alopecuroides* L., seeds were assessed for their anti-HBV repressive activities in an in vitro experimental model using the HepG2.2.15 cell line. The isolated alkaloids were matrine-type alkaloids and showed better inhibitory activities than the positive control lamivudine. Lamivudine (3TC) was able to inhibit the HBV virus to 31.5 percent when its concentration was 1.0 mM while the isolated alkaloids sophaline B and sophaline D significantly repressed the secretion of the HBsAg more than 50 percent. The inhibitory concentrations for these alkaloids against HBV were 0.2 or 0.4 mM. These results suggest that these alkaloids are more potent than the Lamivudine drug [76]. In a separate work by these researchers, another alkaloid called Sophaline-F was identified and showed 53.8% inhibition of the HBsAg secretion at a concentration of 0.035 mM compared to an inhibitory rate of 35.7% for lamivudine. It can be inferred that this alkaloid is more potent than the anti-retroviral drug lamivudine used in this experiment as a positive control [77].

Matrine-type alkaloids derived from another member plant of the *Sophora* genus (*Sophora flavescens* Aiton)*,* also showed more potent anti-HBV activity than Lamivudine drug. Out of all the 17 alkaloids isolated from *Sophora flavescens* Aiton., two of them, flavesine-J and alopecurine-B alkaloids showed significant repressive activities by targeting the HBsAg and HBeAg secretions. The inhibitory concentrations for these alkaloids against HBV were 0.2 mM and 0.4 mM respectively, with an inhibition rate against HBV ranging between 38 and 46% [78].

Berberine alkaloid was reported to have significant anti-HCV activity in Huh-7.5 cells. This virus was repressed by berberine in a dose-dependent way when the concentration of berberine was 1 to 100 μM for 72 h. The EC_50_ concentration for berberine against HCV was 7.87 ± 1.10 μM. An infection assay identified the mode of action of this alkaloid, indicating that berberine strongly blocked HCV entry/fusion. Molecular docking further revealed that berberine interacts with the E2 protein of HCV—an important protein that facilitates viral entry. This targeting of the E2 protein of HCV hinders the entry/fusion process of HCV to the host cell resulting in its inhibition [79].

### Alkaloids against human immunodeficiency virus (HIV)

HIV is a Lentivirus that causes AIDS, a condition in which the immune system gradually weakens, allowing lethal opportunistic infections and malignancies to grow and exacerbate the situation. It is an enclosed RNA virus with a single-stranded, positive-sense genome. Its viral RNA-genome is transformed to double-strand DNA in the infected host cell by reverse transcriptase once it enters the cell [80].

Several alkaloids have been reported to have significant anti-HIV activity. In an in vitro investigation, the bromoindole alkaloid dragmacidin-D obtained from the marine sponge *Halicortex* was tested against HIV. Dragmacidin-D was able to strongly repress the syncytia formation by HIV in the culture cells. These alkaloids also exhibited no cytotoxicity on the culture cells and were able to inhibit this virus at an EC_50_ concentration of EC_50_ of 0.91 μM [81].

Fangchinoline, a bisbenzylisoquinoline alkaloid derived from *Stephania tetrandra* S. Moore (*S. tetrandra*) was found to have antiviral activity in MT-4 and PM1 cells against HIV-1 NL4-3, LAI, and BaL viral strains, with an EC_50_ concentration in the range of 0.8 to 1.7 mM. Fangchinoline had little antiviral action in TZM-b1 cells, but it did inhibit the production of pathogenic virions in HIV-1 cDNA transfected 293T cells, indicating that it is targeting a late event in viral infection. Furthermore, fangchinoline’s antiviral action appears to be envelope-dependent, as it did not affect the generation of pathogenic HIV-1 particles encapsulated with a heterologous envelope. According to a Western blot analysis of HIV envelope proteins generated in transfected 293T cells and isolated virions, fangchinoline lowered HIV-1 gp160 processing, resulting in less envelope glycoprotein integration into nascent virions [82].

Compound SIP-1, an alkaloid compound identified via a compound library screening against HIV, was found to have high repressive activity and lowered the levels of p24, a viral HIV capsid structural protein in macrophages. SIP-1 also showed good binding to the Vpr viral protein of HIV and significant anti-HIV activity. SIP-1 had no negative effects and did not interrupt cell cycle progression or induce apoptosis in Molt-4 and HeLa cell lines, according to MTT, flow cytometry, and a caspase-3 assay. SIP-1 compound also attaches to Vpr specifically, as determined by photo-cross-linked small-molecule affinity beads. SIP-1 reduced viral propagation by more than 98 percent at 10 μM at 8 and 12 days post-infection. At 8 days after infection SIP-1 had an IC_50_ of 0.5 μM against HIV infection. These findings imply that the Vpr protein could be a promising target for the development of HIV-1 replication-blocking medications [83].

6-*O*-Butanoyl castanospermine (B-CAST) alkaloid is a castanospermine alkaloid derivative that has been assessed for its anti-HIV activity via different In vitro experiments. Multiple assays have confirmed that 6-*O*-Butanoyl castanospermine alkaloid is an effective HIV inhibitor. In HeLa T4+ cells and JM cells, HIV-1 induced syncytia formation was potently inhibited by B-CAST with IC_50_ concentrations of 0.3 μg/mL in HeLa T4+ cells assay and an IC_50_ of 0.15 μg/mL in JM cells [84]. The alkaloid castanospermine is also an inhibitor of HIV. It has been reported that castanospermine treatment can reduce the expression of the mature glycoprotein (gp120) of HIV on the host cell surface, with concurrent aggregation of uncleaved precursor gp160, resulting in lower viral infectivity and also repressing the HIV-induced syncytium formation in the cultured cells [85].

The manzamine-based novel alkaloids manadomanzamine A, B, and xestomanzamine-A were isolated from the marine sponge *Acanthostrongylophora* sp., and exhibit significant anti-HIV activities in PBM cells. These alkaloids were found to have EC_50_ values of 7.0, 16.5, and 11.2 g/mL against human immunodeficiency virus, respectively [86].

Emetine, an ipecac alkaloid, has significant anti-HIV properties. Emetine can lower HIV infection in both non-lymphocytic and peripheral blood mononuclear cells by up to 80 percent. Reverse transcriptase and NERT assays showed that emetine has strong inhibitory activity against the HIV reverse transcriptase. The NERT assay results indicated that emetine directly penetrates the viral HIV particles and inactivates its reverse transcriptase enzyme activity with an inhibitory rate of 90% at a concentration of 14.1 mM. These findings imply that emetine alkaloid could be employed as a possible microbicide against HIV-1 [87].

Aside from these, other manzamine-based alkaloids have considerable anti-HIV efficacy. Manzamine-A, neokauluamine, 8-hydroxymanzamine-A, 6-deoxymanzamine-X, and 12,28-oxamanamine-A alkaloids have been reported to have inhibitory activity with EC_50_ values of 4.2 µM, 2.3 µM, 0.59 µM,1.6 µM, and 22.2 µM against HIV [88, 89]. Along with these alkaloids, Tulongicin based alkaloids have anti-HIV activity. These tulongicin alkaloids were extracted from the *Topsentia* sp., a sponge of deep waters. These alkaloids (tulongicin-A and dihydrospongotine-C) had an IC_50_ concentration of 3.9 μM and 2.7 μM against the YU2-HIV isolate respectively, while their IC_50_ concentrations against the HxB2-HIV isolate was 3.5 μM and 4.5 μM respectively [90].

Alkaloids extracted from the tropical *Ancistrocladus korupensis* Thomas and Gereau—1993 have been reported as anti-HIV inhibitors. These alkaloids michellamines D and F showed significant repressive activity against multiple strains of HIV. These alkaloids prevented the cytopathic effect of HIV in the CEM-SS cell line. Michellamines D and F exhibited significant cytoprotection against HIV with EC_50_ dosages of 3 μM and 2 μM against the HIV-RF strain respectively [91]. Triptonine B, a sesquiterpene alkaloid, has significant anti-HIV action. In in vitro evaluation investigations, this alkaloid demonstrated inhibitory efficacy against HIV with an EC_50_ of 0.10 μg/mL. Its therapeutic index was also greater than 1000, indicating that this alkaloid has a high potential for development as an anti-HIV medication [92].

### Alkaloids against middle east respiratory syndrome coronavirus (MERS-CoV) and human coronavirus OC43 (HCoV-OC43)

MERS-CoV and HCoV-OC43 are enveloped coronaviruses (CoVs) with positive-sense ssRNA as genetic material. These viruses are zoonotic and jump from animals to human hosts. They cause multiple respiratory infections. The most common subtype of HCoV, HCoV-OC43, is responsible for over 30% of respiratory infections and can cause re-infection for the rest of one’s life [93].

An in vitro investigation found three alkaloids with anti-HCoV-OC43 potential in MRC-5 cells: tetrandrine (TET), fangchinoline (FAN), and cepharanthine (CEP). In MRC-5 cell culture, treatment with these three alkaloids reduced the cytopathic effect of HCoV-OC43. The IC_50_ concentrations which reduced the cytopathic effect of these alkaloids were 0.33 ± 0.03 μM,1.01 ± 0.07 μM, and 0.83 ± 0.07 μM respectively. Time of addition assays revealed that the pre and co-treatment of these alkaloids effectively reduced the infection in MRC-5 cells and these alkaloids likely target the early stages of viral infection. Further investigation revealed that these alkaloids decreased viral RNA copies. The expression levels of the HCoV-OC43 N and S-proteins were also lowered after treatment with these alkaloids. Moreover, treatment with these alkaloids also attenuated the virus-induced host response. These results indicate a multi-stage inhibition of this virus by these three alkaloids [94]. In an in vitro screening assay (MERS-ELISA assay), multiple compounds had repressive MERS-CoV potential. This investigation reported an alkaloid, emetine dihydrochloride hydrate, to have a significant inhibitory effect against MERS-CoV, with an EC_50_ value of 0.014 M. Moreover, with an EC_50_ value of 0.051 M, this alkaloid also suppressed SARS-CoV [95].

### Alkaloids against the respiratory syncytial virus (RSV)

RSV is an orthopneumovirus with ssRNA as genetic material. The most typical way for the virus to spread is through close contact with saliva or mucus droplets. RSV is one of the most prevalent viruses that cause respiratory diseases in newborns, the elderly, and those with weakened immune systems, resulting in substantial infection rates worldwide each year [96].

A comprehensive study reported that cyclopamine (CPM) alkaloid and its chemical analogue A3E have potent anti-RSV activities. These alkaloids were first evaluated in an in vitro study performed in Hep-2 cells on different RSV clinically isolated strains. This study reported that the RSV strains RSV-A GA2, RSV-A ON1, and RSV-B were potently inhibited by both the CPM and A3E alkaloids. The IC_50_ concentration of CPM against these RSV strains was 0.82 μM, 0.46 μM, and 0.76 μM and that of A3E which is cyclopamine analogue were 2.92 μM, 3.00 μM, and 3.72 μM respectively. Moreover, these alkaloids were evaluated in mice, and both compounds showed significant anti-RSV activities by targeting the replication process of RSV. These results suggest a strong antiviral efficacy of alkaloids both in vitro and in vivo studies [97].

A patent publication reports several indole alkaloid-based compounds having significant anti-RSV activities. These compounds had EC_50s_ ranging from 0.0007 to 0.512 μM, and thus are promising inhibitory compounds for RSV. These inhibitors are reported to be fusion protein inhibitors of RSV and target the entry and attachment of RSV to host cells [98].

### Alkaloids against parainfluenza virus-3 (PI-3) and Sindbis virus (SINV)

Human parainfluenza viruses (HPIVs) are members of the paramyxoviridae virus family and are a common cause of upper and lower respiratory illnesses. The PI-3 virus virion contains negative-sense ssRNA. Although most HPIV illnesses are minor and self-limiting, serious infections that require hospitalisation can also happen, especially in newborns, youngsters, and people with impaired immune systems [99]. SINV is an enclosed RNA virus in the genus Alphavirus. Sindbis virus is related to the Chikungunya alphavirus. *Culex* mosquitos spread the Sindbis virus to a range of birds, including migratory and game birds, which act as a carrier of this virus. *Culex* and, less frequently, *Aedes* mosquitos transmit the virus to dormant hosts like humans, and it is a recurrent cause of polyarthritis, rash, and fever, however many infections are asymptomatic. Karelian fever is another clinical condition linked to SINV infection [100].

In a study on the effects of different phytochemicals including flavonoids, plant phenolics, and alkaloids, two alkaloids had a potent antiviral effect on PI-3. Atropine and octopamine alkaloids showed good antiviral activity on PI-3 in the MDBK cell line. Alkaloids tested in this experiment showed significant inhibition and reduction of the cytopathogenic effect (CPE) caused by the Human parainfluenza virus-3. Both atropine and octopamine alkaloids inhibited the virus with a maximum inhibitory concentration of 0.05 µg/mL [25]. Deoxynojirimycin and castanospermine alkaloids have been shown to exhibit antiviral action against the Sindbis virus in BHK cells. These alkaloids inhibited virion assembly by acting on a step in the viral glycoprotein processing. An asparagine amino acid residue in the glycoprotein of this virus has a linked oligosaccharide moiety (GlcNAc_2_, Man_9_, Glc_3_). The removal of glucose from this oligosaccharide moiety starts further processing of this viral protein and is critical for attaining its correct functional conformation. These two alkaloids blocked this step by acting on the glucosidase-I enzyme, resulting in the inhibition of the Sindbis virus protein assembly. Moreover, a precursor glycoprotein (PE2) which upon cleaving forms the glycoprotein-(E2) and is essential for the virion formation was also inhibited by these two alkaloids by interfering with and blocking the cleavage of PE2 into E2 glycoprotein [101].

### Alkaloids against infectious bursal disease virus (IBDV) and porcine epidemic diarrhoea virus (PEDV)

Both of these viruses cause diseases in animals. IBDV is a retrovirus that can cause a highly contagious disease in young poultry, causing bursal necrosis and severe immune system impairment [102], while PEDV is an Alphacoronavirus and the primary cause of acute diarrhea, dehydration, and significant mortality in pigs [103, 104].

Several cyclopeptide alkaloids conjugates were extracted from *Ziziphus jujuba* Mill., the plant was evaluated against PEDV and it showed strong inhibitory activities in the in vitro antiviral assays. Jubanine-G, Jubanine-H, and Nummularine-B alkaloids potently inhibited PEDV infection in Vero cells. The EC_50_ inhibitory concentrations for these alkaloids against this virus were 13.41 ± 1.13 μM, 4.49 ± 0.67 μM, and 6.17 ± 0.50 μM respectively. These alkaloids were also non-toxic to the tested cell line [105]. Another alkaloid—homoharringtonine (HHT)—has been reported as an anti-PEDV agent in vitro and in vivo. HHT treatment of PEDV-infected Vero cells showed reduced viral loads at a concentration of 200 nM, and at 500 nM HHT concentration the infection was completely suppressed. The IC_50_ value of HHT against PEDV was 0.112 µM and it resulted in a significant drop in N protein and mRNA levels of this virus. In the in vivo studies, HHT was efficient at suppressing PEDV viral production in piglets and hens at doses of 0.05 mg/kg and 0.2 mg/kg, respectively. All of the animals given the treatment survived with no pathogenic alterations in their tissues or symptoms of infection [30].

*C. metuliferus* Emey., fruit alkaloids have been reported to be anti-IBDV active in chicken fibroblast cells. These alkaloid extracts showed antiviral activity at a concentration of 6.125 and 100 mg/mL. The positive control group showed cytopathic effects, whereas the negative control group treated with alkaloids showed no cytopathic effects [106].

### Alkaloids against bovine viral diarrhea virus (BVDV)

BVDV is a Pestivirus. It can be found in the majority of countries throughout the world [107], causing mucosal diseases, respiratory and GI-tract infections, and reproductive complications in cattle [108].

Alkaloid 6-*O*-butanoyl castanospermine (celgosivir), a derivative of castanospermine, was evaluated against the BVDV in different in vitro studies. Celgosivir exerted strong anti-BVDV activities and inhibited the release and mRNA production of this virus. Its inhibitory activity was measured in both plaque reduction and CPE assays. Celgosivir repressed BVDV growth at an IC_50_ of 16 µM and 47 µM respectively. Mechanism of action studies revealed that celgosivir repressed the viral RNA and also reduced the quantity of the viral infectious units released from the infected cells. This alkaloid also repressed BVDV E2 viral protein expression levels. Moreover, the combination of this alkaloid separately each with Ribavirin and interferon-α showed a synergistic relationship in the inhibition of BVDV in plaque reduction assays [109].

The alkaloid *n*-butyl deoxynojirimycin (*n*B-DNJ) was evaluated in another In vitro study for its synergistic effects when used in combination with interferon-α (IFN) against BVDV. *n*B-DNJ is a reported glucosidase repressor and inhibits viral production by compromising viral protein folding and thereby limiting and inhibiting viral assembly. In this study, it was used with IFN to study its effect on BVDV. It has been reported that IFN alone in plaque reduction and cytopathic assays reduces viral growth with an IC_50_ of 3 IU/mL. When IFN was used in combination with 138 µM of *n*B-DNJ alkaloid the IC_50_ of IFN decreased to 0.056 IU/mL and its antiviral potency increased 54-fold against BVDV. These results demonstrate that this alkaloid can synergistically increase the BVDV viral inhibition when used with IFN in combinational therapy [110].

### Alkaloids against coxsackieviruses (CV)

CVs are enteroviruses that cause ailments varying from gastrointestinal distress to pericarditis and myocarditis (CVB3-induced cardiomyopathy) [111], CVB3 thrives in the myocardium and produces severe coronary illnesses [112].

Numerous matrine-type alkaloids and crude alkaloids from *S. tonkinensis* Gagnip., have been found to have substantial anti-CVB3 properties. These extracted alkaloids were evaluated in Vero cells for antiviral activity against CV B3. Out of these extracted alkaloids, the most potent was (−)-12β-hydroxyoxysophocarpine alkaloid. This alkaloid showed an IC_50_ of 26.62 µM against the coxsackievirus B3. In comparison to the positive control ribavirin (RBV) (IC_50_ of 1197.58 µM), the (−)-12-hydroxyoxysophocarpine alkaloid showed 45 times greater anti-CVB3 activity, with a therapeutic selectivity index also equivalent to RBV [113].

Aranotin and gliotoxin are sulfur-containing alkaloids that have been also reported as anti-CV A21. Aranotin was found to be effective against CV A21 in the In vivo mice model, with an ED_50_ of 0.125 mg/kg i.p. [114]. Gliotoxin alkaloid is active against a broad range of RNA viruses including the CV and inhibits these viruses by targeting their RNA synthesis [115]. Another common alkaloid, caffeine, is also been found to inhibit the growth of CV [116].

### Alkaloids against murine leukemia virus (MLV)

MLVs are Gammaretroviruses that cause malignancy in their murine (mouse) hosts. Other vertebrates may be infected by some MLVs. MLVs have a positive-sense ssRNA genome that replicates by reverse transcription via a DNA intermediary [117].

6-*O*-Butanoylcastanospermine (B-CAST) is a castanospermine alkaloid that was evaluated for its anti-MLV activities in chronically infected C3HlOTY1/2 (clone 8) cells. This analog of castanospermine was more potent than the other analogues prepared in this study. As B-CAST is also a glucosidase inhibitor, this enzyme causes the misfolding of viral proteins and strongly represses the activity of the MLV in plaque reduction assay. MLV was inhibited by CAST-B with an IC_50_ of 0.05 µg/mL. CAST-B also showed strong inhibition of the glucosidase enzyme with an IC_50_ of 0.7 µg/mL. Viral replication of MLV was targeted by this alkaloid and a relationship between glucosidase-I inhibition and MOLV replication was observed in this experiment [84]. Like the previously discussed CAST-B alkaloid, castanospermine and 1-deoxynojirimycin alkaloids are effective inhibitors of glucosidases I and II. These two alkaloids demonstrated substantial potency against the Moloney murine leukemia virus in separate research, with an inhibitory concentration of (IC_50_: 1.2 g/mL). These findings imply that glucosidase enzymes are required for the replication of MLV and that targeting these enzymes by these alkaloid inhibitors via a unique mechanism of action could lead to the development of chemopreventive and therapeutic medicines against MLV infections [118].

### Alkaloids against vesicular stomatitis virus (VSV)

VSV is a zoonotic arbovirus that is related to rabies viruses and belongs to the Rhabdoviridae family. VSV contains an 11-kb genome made up entirely of negative-sense RNA. VSV is transmitted to animals through bug bites, and in cattle, horses, and swine, and can cause severe disease with symptoms comparable to foot and mouth disease. Human infections of this virus also occur, but they are far less common than in animals, and the symptoms are much milder (typically mild flu-like symptoms). Many infections in people go unnoticed because they are asymptomatic; but, on rare occasions, serious illness has also been documented [119, 120].

Homoharringtonine (HHT) alkaloid has been reported to have dose-dependent antiviral activity against VSV. In this study, VSV was inoculated into HEK293T cells in the presence of increasing concentrations of HHT or ribavirin. When cells were treated with HHT at 50 nM concentration, the viral output of VSV productions was reduced by 1.5 orders of magnitude, and viral yields were drastically reduced when cells were treated with HHT at 100 nM concentration. There was no discernible decline in cell viability in the presence of these tested HHT concentrations. Ribavirin was found to have antiviral action at doses greater than 100 nM which suggests that HHT is more potent in the inhibition of VSV than Ribavirin. HHT treatment resulted in a significant reduction in viral protein G levels of VSV at 24 and 36 h after infection (h.p.i.). To determine the inhibitory stage of HHT, time-of-addition tests were performed, and it was discovered that HHT does not block VSV cell entry, and targets a late stage of VSV viral replication thereby inhibiting it [30].

### Alkaloids against Newcastle disease virus (NDV)

NDV is a zoonotic virus that infects all species of birds and is a member of the paramyxoviridae family. NDV has a negative-sense ssRNA that is roughly 15 kb long and nonsegmented. NDV causes infections in poultry farming birds, and it is a persistent threat to the poultry industry all over the world [121]. According to some studies, NDV is nonpathogenic in primates [122], however, a human case of deadly meningoencephalitis caused by NDV has also been documented recently [123].

The antiviral properties of emetine have been investigated against a large number of RNA/DNA viruses and have been found to have substantial antiviral activity both in the in-ovo and in vitro. At a non-toxic dose of 200 nM, emetine treatment reduced viral RNA synthesis from NDV and prevented viral entry into host cells at the time of addition and in virus step-specific tests. In addition, emetine therapy resulted in a decrease in viral protein synthesis. Emetine was found to severely limit NDV replication in a cell-free endogenous virus polymerase assay. This suggests that in addition to directly blocking certain viral polymerases, emetine can target additional components that are important for successful viral genome replication. Emetine has also been found in in-ovo studies to dramatically delay NDV-induced death in chicken embryos by reducing the NDV virus titers [124].

The antiviral effect of another alkaloid—homoharingtonin (HHT) was investigated for its antiviral activity in both in vitro and in-ovo test models. It was revealed that GFP-NDV-infected cells were inhibited dose-dependently. Light microscopy showed a substantial reduction in the generation of recombinant NDV expressing green fluorescent protein (GFP) in the presence of HHT at 50 nM. When compared to mock-infected samples, cultured HeLa cells showed no difference in morphology or number when treated with 100 nM HHT. The infection rate was reduced in a dose-dependent manner by HHT therapy. The inhibition concentration (IC_50_) value for HHT was 18 nM against NDV. Furthermore, no detectable loss in cell viability was detected at HHT doses less than 1 µM. HHT also exerted a strong inhibitory effect on NDV by lowering the level of NDV-NP protein expression in cells treated with 50 nM HHT. In ovo and in vivo studies, showed potent activity of HHT against NDV. The best inhibitory activity against NDV in the in-ovo studies on chicken embryos was 0.2 mg/kg HHT. In the in vivo studies performed on chickens, HHT at 0.2 mg/kg significantly reduced NDV-NP mRNA levels in the liver, lungs, and blood. There were no pathological changes in tissues or symptoms like diarrhea in HHT-treated animals. HHT was efficient in its anti-NDV activity, and it acted against NDV by lowering the viral load in NDV-infected cells, embryos, and animals. In this work, ribavirin reduced GFP-NDV DNA synthesis with an IC_50_ of 44.241 µM, which is higher than the IC_50_ of HHT, which is 0.018 µM. These experiments reveal that HHT therapy was more effective than ribavirin at the recommended dosages, without impacting cell viability [30].

### Alkaloids against poliovirus (PV) and enterovirus 71 (EV71)

PV is a picornavirus in the enterovirus C family. It is the virus that causes polio. PV comes in three different variants and has an RNA genome and a protein capsid. Its genome is made up of positive-sense ssRNA and measures approximately 7.5 kb in length [125]. Enterovirus 71 (EV71) is also a picornavirus belonging to the genus *Enterovirus* and is known to cause severe neurological diseases as well as hand-foot-and-mouth disease (HFMD) in young people [126, 127].

Lycorine alkaloid, isolated from *C. miniata* (Lindl.) regel (family Amaryllidaceae) showed anti-PV activity in vitro. It has been reported that lycorine alkaloid showed significant inhibitory activities in Vero cells against PV by reducing the CPE caused by this virus. The viral CPE of poliovirus on VERO cells was strongly suppressed at doses of 2.5 µg/mL [128]. Aporphinoid alkaloids (Glaucine fumarate, *N*-methyllaurotetanine, and isoboldine-HCl) have also been reported to have anti-PV activities. At effective dosage (ED_50_) of glaucine fumarate 9 µM and *N*-methyllaurotetanine, isoboldine-HCl both at 15 µM repressed the Poliovirus by suppressing the CPE caused by it in the yield reduction assays [129]. Other alkaloids, such as pericalline, Pervine along with periformyline and leucristine have also been found to have anti-PV properties [130]. In vitro and in vivo mouse model investigations have shown that lycorine alkaloid is also anti-EV-71. It has been reported that lycorine acts on limiting viral multiplication, and also diminishes the CPE on rhabdomyosarcoma (RD) cells with an IC_50_ dosage of 0.48 μg/mL. According to the mode of action studies, it represses viral EV-71 protein synthesis. Further analysis also showed that it inhibits the viral polyprotein from elongating during the translation process. Lycorine therapy of mice challenged with a fatal dose of EV71 resulted in a reduction in mortality and degenerative changes in the muscles of the mice. Lycorine therapy at 0.4 or 1.0 mg/kg resulted in a 45 percent improvement in mouse survival; also, Lycorine therapy protected mice against paralysis after infection with a moderate dose of EV71 [131].

### Alkaloids against West Nile virus (WNV) and rabies virus (RABV)

West Nile virus is an ssRNA virus and the causative agent of West Nile fever. It belongs to the Flaviviridae family, including the Zika virus, DENV, and yellow fever virus. Mosquitoes, especially *Culex pipiens*, are the major carriers of the virus [132]. In contrast, the Rabies virus (rabies lyssavirus) is a neurotrophic virus that causes rabies in humans and animals. Rabies can be spread by animal saliva and, in rare cases, through contact with human saliva. Rabies lyssavirus, like many other rhabdoviruses, has a diverse host range. Many mammal species have been documented to be infected with RABV in the wild [133]. Rabies lyssavirus spreads fast through the peripheral nervous system`s neural pathways after entering through the wound. During natural infection, rabies lyssavirus retrograde axonal transport to the central nervous system (CNS) is a critical phase in pathogenesis [134]. Although binding of the rabies P protein to the dynein light chain protein DYNLL1 has been demonstrated, the specific molecular mechanism of its transport is unknown. P-protein also functions as an interferon antagonist, lowering the host's immunological response [135]. It has been reported that emetine is active against this discussed pathway of infection of RABV in the N-compartment axons. Infected cells pretreated with emetine showed a dose-dependent reduction in RABV infection. Twenty-four hours after infection, the proportion of infected cells in the untreated state was 29.4 ± 8.5%, compared to 14.0 ± 6.7%, 4.5 ± 2.0%, 0.01 ± 0.02% under 10 μM, 50 μM, or 100 μM emetine treated ones. As illustrated above, the retrograde infection of RABV was essentially stopped at 24 hpi in axons treated with 100 μM emetine. These findings add to our knowledge of how neuroinvasion is controlled in axons and suggest that emetine alkaloid may play a role as an inhibitory modulator of RABV axonal transport [136]. The alkaloid palmatine has been reported to be active against WNV in Vero cells. Palmatine can suppress WNV without cytotoxicity and reduces virus titers by a factor of 44 at 100 µM in plaque assays. The EC_50_ of palmatine measured for WNV was 3.6 μM. Palmatine inhibited WNV by targeting NS2B-NS3 protease activity [137]. A summary of the antiviral activities of some of the above-discussed alkaloids is given below (Table 1).

**Table 1:** Antiviral activities of the discussed alkaloids

S. no.	Viruses	Alkaloid	Inhibitory concentrations/binding energies	Experimental model	Inhibitory stage/target or mechanism	Ref.
1	Influenza virus	Berberine	IC_50_ = 0.025 g/L and 0.005 g/(kg/day)	In vitro and in vivo	Protein-transport and maturation	[19]
		Homonojirimycin (HNJ)	EC_50_ = 10.4 μg/mL	In vitro	Host immune system modulation	[20]
		Oxoglyantrypine	IC_50_ = 85 μM	In vitro	Host immune system modulation	[21]
		Norquinadoline A	IC_50_ = 82 μM	In vitro		
		Deoxynortryptoquivaline	IC_50_ = 87 μM	In vitro	Unknown	[22]
		Deoxytryptoquivaline	IC_50_ = 85 μM			
		Tryptoquivaline	IC_50_ = 89 μM			
		Compound-14	IC_50_ = 17.9 ± 2.0 μM	In vitro	Unknown	[23]
2	Herpes simplex virus	Yohimbine	0.8 µg/mL	In vitro	Repression in the CPE	[25]
		Vincamine	0.8 µg/mL			
		Atropine	0.8 µg/mL			
		Trigonelline	0.4 µg/mL			
		Allantoin	1.6 µg/mL			
		Octopamine	1.6 µg/mL			
		Synephrine	1.6 µg/mL			
		Colchicine	1.6 µg/mL			
		Manzamine-A	IC_50_ = 5.6 µM	In vitro	Viral transcription	[26]
		Tetrandine	50 mg/kg	in vivo	Host immune system modulation	[27]
		6-*O*-Butanoyl castanospermine	IC_50_ = 15 ± 4.8 µM	in vivo	Reduced viral load in brain tissue	[28]
		Homoharringtonine	IC_50_ = 139 nM	In vitro	Viral protein (ICP-8) synthesis	[29]
		Tubingensin A	IC_50_ of 8 µg/mL	In vitro	Unknown	[31]
		Berberine	IC_50_ = 8.2 ± 1.2 × 10^–2^ mg/mL	In vitro	Viral protein gB and gE synthesis	[32]
3	Dengue virus	Cherylline	EC_50_ = 8 μM	In vitro	Viral replication	[37]
		Anisomycin	200 nM	In vitro	RNA synthesis	[38]
		Castanospermine	IC_50_ = 85.7 μM(BHK-21) and 1 μM (Huh-7)	In vitro	Viral protein (prM-E) and (C) incorporation into the Virus	[39]
		Tomatidine	EC_50_ = 0.82–4.87 μM	In vitro	Viral-E protein synthesis	[40]
		3-Oxo-voacangine Voacangine-7-hydroxyindolnine Rupicoline Voacangine	Unknown	In vitro	Virucidal effect	[41]
4	Chikungunya virus	Berberine	EC_50_ = 1.8 μM	In vitro	Protein synthesis and RNA synthesis	[43]
		Harringtonine	EC_50_ = 0.24 μM	In vitro	Protein synthesis and RNA synthesis	[16]
		Tomatadine	EC_50_ = 1.3 μM	In vitro	Post entry stages	[44]
5	Ebola virus	Emetine	IC_50_ of 10.2 μM and 1 mg/kg/day	In vitro and in vivo	Viral entry	[47]
		Piperine	− 7.1 to − 5.8 kcal/mol	in silico	Inhibition of EBOV Nsps	[48]
6	Human Cytomegalo-virus	Emetine	EC_50_-40 ± 1.72 nM and 0.1 mg/kg	In vitro and in vivo	Post viral-entry stages	[49]
		Berberine chloride	IC_50_ = 0.68 μM	In vitro	Post entry stages	[50]
7	Zika virus	Cherylline	EC_50_ = 20.3 μM	In vitro	Replication	[37]
		Lycorine	EC_50_ = 0.41 μM			
		Anisomycin	EC_50_ = 7.9 ± 1.2 nM	In vitro	Initial and intermediate stages Protein synthesis and Replication	[38]
		Emetine	IC_50_ = 52.9 nM			
		Fusaindoterpene-B	EC_50_ = 7.5 nM			
		JBIR-03	EC_50_ = 4.2 μM	In vitro	Unknown	[58]
		1,2-bis(1*H*-Indol-3-yl) ethane-1,2-dione	EC_50_ = 5.0 μM			
		Cephaeline	5 mg/kg/day	In vivo	Viral RNA synthesis	[47]
		Palmatine	10 to 80 μM	In vitro	Multi-stage inhibitor	[59]
8	SARS-CoV-2	Ajmalicine	− 8.1 kcal/mol	In silico	Replication	[66]
		Aranotin	− 8.2 kcal/mol			
		Chloroquine	EC_50_ = 2.1 μM	In vitro	Replication	[67]
		Hydroxy-chloroquine	EC_50_ = 1.5 μM			
		Pyronaridine	EC_50_ = 1.82 μM			
		Homorringtonine	EC_50_ = 2.55 mΜ	In vitro	Replication	[72]
		Emetine	EC_50_ = 0.46 μM			
9	Hepatitis-B virus	5,6-Dihydro-6-methoxynitidine	0.2 μmol/mL	In vitro	Targeted the secretion of HBsAg and HBeAg viral HBV-proteins	[75]
		Skimmianine	0.2 μmol/mL			
		5-Methoxydictamine	0.2 μmol/mL			
		Sophaline B	0.4 mM	In vitro	Targeted the secretion of HBsAg and HBeAg viral HBV-proteins	[76]
		Sophaline D	0.4 mM			
		Sophaline C	0.2 mM			
		Flavesine-J	0.2 mM	In vitro	Targeted the secretion of HBsAg and HBeAg viral HBV-proteins	[77]
		Alopecurine-B	0.4 mM			
10	Hepatitis C virus	Berberine	EC_50_ = 7.87 ± 1.10 μM	In vitro	Viral entry/fusion	[79]
11	HIV	Dragmacidin-D	EC_50_ = 0.91 μM	In vitro	Inhibited viral syncytia formation	[81]
		Fangchinoline	EC_50_ = 0.8 to 1.7 mM	In vitro	Late stages of infection	[82]
		SIP-1	IC_50_ = 0.5 μM	In vitro	Replication	[83]
		6-*O*-Butanoyl castanospermine	IC_50_ = 0.15 μg/mL	In vitro	Inhibited viral syncytia formation	[84]
		Castanospermine	–	In vitro	Glycoprotein synthesis repressor	[85]
		Manadomanzamine A	EC_50_ = 7.0 g/mL	In vitro	Unknown	[86]
		Manadomanzamine B	EC_50_ = 16.5 g/mL			
		Xestomanzamine-A	EC_50_ = 11.2 g/mL			
		Emetine	14.1 mM	In vitro	HIV reverse transcriptase	[87]
		Manzamine-A	EC_50_ = 4.2 µM	In vitro	Unknown	[88, 89]
		Neokauluamine	EC_50_ = 2.3 µM			
		8-Hydroxymanzamine-A	EC_50_ = 0.59 µM			
		6-Deoxymanzamine-X	EC_50_ = 1.6 µM			
		12,28-Oxamanamine-A	EC_50_ = 22.2 µM			
		Tulongicin-A	IC_50_ = 3.9 μM	In vitro	Unknown	[90]
		Dihydrospongotine-C	IC_50_ = 2.7 μM			
		Michellamines D	EC_50_ = 3 μM	In vitro	Unknown	[91]
		Triptonine B	EC_50_ of < 0.10 μg/mL	In vitro	Unknown	[92]
		Michellamines F	EC_50_ = 2 μM	In vitro	Unknown	[91]
12	HCoV-OC43	Tetrandrine	IC_50_ = 0.33 ± 0.03 μM	In vitro	Multi-stage inhibitor	[94]
		Fangchinoline	IC_50_ = 1.01 ± 0.07 μM			
		Cepharanthine	IC_50_ = 0.83 ± 0.07 μM			
13	MERS-CoV	Emetine	EC_50_ = 0.014 μM	In vitro	Viral entry	[95]
14	SARS-CoV	Emetine	EC_50_ = 0.051 μM	In vitro	Viral entry	[95]
15	Respiratory syncytial virus	Cyclopamine	IC_50_ = 0.46 to 0.82 μM	In vitro	Viral entry	[97]
		A3E	IC_50_ = 2.92 to 3.72 μM			
16	Parainfluenza virus-3	Atropine	0.05 µg/mL	In vitro	Reduction in the CPE	[25]
		Octopamine	0.05 µg/mL			
17	Sindbis virus	Deoxynojirimycin	–	In vitro	Viral glycoprotein processing	[101]
		Castanospermine	–			
18	Infectious bursal disease virus	Alkaloid extracts	6.125 and 100 mg/mL	In vitro	Reduction in the CPE	[106]
19	Porcine epidemic diarrhoea virus	Jubanine-G	EC_50_ = 13.41 ± 1.13 μM	In vitro	Replication	[105]
		Jubanine-H	EC_50_ = 4.49 ± 0.67 μM			
		Nummularine-B	EC_50_ = 6.17 ± 0.50 μM			
		Homoharringtonine	IC_50_ = 0.112 µM and 0.2 to 0.05 mg/kg	In vitro and in vivo	mRNA and protein synthesis	[30]
20	Bovine viral diarrhea virus	6-*O*-Butanoyl castanospermine	IC_50_ = 16 µM	In vitro	Viral RNA synthesis process	[109]
		*n*-Butyl deoxynojirimycin	138 µM	In vitro	Viral glycoprotein processing	[110]
21	Coxsackievirus	(−)-12β-Hydroxyoxysophocarpine	IC_50_ = 26.62 µM	In vitro	Unknown	[113]
		Aranotin	ED_50_ = 0.125 mg/kg	In vitro	Unknown	[114]
		Gliotoxin	–			[115]
22	Murine leukemia virus	6-*O*-Butanoylcastanospermine	IC_50_ = 0.05 µg/mL	In vitro	Viral replication	[84]
		Castanospermine	IC_50_ = 1.2 g/mL		Viral glycoprotein processing	[118]
23	Vesicular stomatitis virus	Homoharringtonine	100 nM	In vitro	Viral replication	[30]
24	Newcastle disease virus	Emetine	200 nM	In ovo	Multi-stage inhibitor	[124]
		Homoharingtonin	IC_50_ = 18 nM	In vitro	mRNA synthesis	[30]
25	Polio virus	Lycorine	2.5 µg/mL	In vitro	Reduction in the CPE	[128]
		Glaucine fumarate	ED_50_ = 9 µM	In vitro	Reduction in the CPE	[129]
		*N*-Methyllaurotetanine	ED_50_ = 15 µM			
		Isoboldine-HCl	ED_50_ = 15 µM			
26	Enterovirus 71	Lycorine	IC_50_ = 0.48 μg/mL and 0.4 mg/kg	In vitro and in vivo	Viral protein synthesis	[131]
27	West Nile virus	Palmatine	EC_50_ = 3.6 μM	In vitro	Reduction in viral titers	[137]
28	Rabies virus	Emetine	10 to 100 μM	In vitro	Viral axonal transport	[136]

The effective dosage 50 (**ED**_**50**_) is a pharmacological phrase that refers to the amount of medicine that induces a therapeutic response or intended effect in 50% of the patients that take it. In a pharmacological context, this can be the drug concentration required to provide half of the maximum feasible effect. The half-maximal inhibitory concentration (**IC**_**50**_) of a medication is a measure of its efficacy. It denotes the amount of the drug required to inhibit a biological process by half. While binding free energy is the total of all intermolecular interactions between the ligand and the target

## Comparison of some antiviral alkaloids and standard drugs along with a brief overview of their toxicological effects and therapeutic uses

The alkaloids discussed here showed significant antiviral activities against different viruses, sometimes more potent than standard treatments. The dosage required for alkaloids to inhibit these viruses compared to the positive control drugs was also lower than the standard drugs. Confirming that alkaloids can be more potent in their repressive activities against different viruses. Against HBV, different alkaloids demonstrated more potent activities than the standard drug lamivudine, with lower inhibitory concentrations and higher inhibitory rates. Harringtonine also showed better antiviral activities than the antiviral drugs Rottlerin and Ribavirin, and at much lower inhibitory concentrations. Similarly, Berberine was noted to have higher inhibitory activity with an IC_50_ = 0.025 g/L compared to the Ribavirin IC_50_ = 0.051 g/L concentration, capsaicin alkaloid also showed lower IC_50_ values against the Para-influenza virus compared to the Oseltamivir which is a standard antiviral drug, this similar trend of higher activity at lower IC_50_ concentrations were also seen with a few other antiviral alkaloids against different viral targets. We also discovered that some of the reviewed alkaloids here were also able to boost the activity of the standard antiviral drugs when used in combination therapy against different viruses. A comparison of some alkaloids and standard antiviral drugs is given in Table 2*.*

**Table 2:** Comparison of antiviral activities of alkaloids and standard drugs

S.N	Virus	Alkaloid	Inhibitory concentration	Standard drug	Inhibitory concentration	Ref.
1	HBV	5,6-Dihydro-6-methoxynitidine	0.2 μmol/mL	Lamivudine	1.0 μmol/mL	[75]
2	HBV	5-Methoxydictamnine	0.2 μmol/mL	Lamivudine	1.0 μmol/mL	[75]
3	CHIKV	Harringtonine	EC_50_ = 0.24 μM	Rottlerin	More than 10 μM	[16]
4	CHIKV	Harringtonine	EC_50_ = 0.24 μM	Ribavirin	EC_50_ = 2.05 μM	[16]
5	HSV	Yohimbine	0.8 µg/mL	Acyclovir	1.6 µg/mL	[25]
6	HSV	Vincamine	0.8 µg/mL	Acyclovir	1.6 µg/mL	[25]
7	HSV	Atropine	0.8 µg/mL	Acyclovir	1.6 µg/mL	[25]
8	HSV	Trigonelline	0.4 µg/mL	Acyclovir	1.6 µg/mL	[25]
9	PI-3	Capsaicin	0.2 µg/mL	Oseltamivir	1.6 µg/mL	[25]
10	PI-3	Allantoin	0.4 µg/mL	Oseltamivir	1.6 µg/mL	[25]
11	CV-B3	(−)-12-Hydroxyoxysophocarpine	IC_50_ = 26.62 µM	Ribavirin	IC_50_ = 1197.58 µM	[92]
12	IV	Berberine	IC_50_ = 0.025 g/L	Ribavirin	IC_50_ = 0.051 g/L	[19]

Despite their known antiviral benefits, several alkaloids are recognized as excessively harmful and are not approved to be administered to individuals or animals. but despite its reported toxicity like other drugs the toxicity of Alkaloids also depends on the doses administered to the test subjects. Apart from its reported toxicity alkaloid compounds have been utilized in treating various diseases the alkaloid homoharringtonine (HHT) reported here is used for treating chronic myeloid leukemia [138]. Moreover, several alkaloids are being utilized in the Management of Diabetes Mellitus [139]. Other than these alkaloids are actively being utilized for ages for treating malarial infections [140] and are a broad source of medicinal compounds. Furthermore, due to the scarcity of detailed data on the toxic effects of the investigated alkaloid compounds in clinical research on human subjects, it is challenging to make inferences concerning the general relevance and utilization of alkaloid phytochemicals. Moreover, the administration of these alkaloids in different research settings conducted on these substances included in this study did not show any negative side effects in the in-vitro, in-vivo, or in-ovo investigations reviewed here, so based on these observations alkaloid compounds can be considered as promising anti-viral agents.

**Fig. 2:**
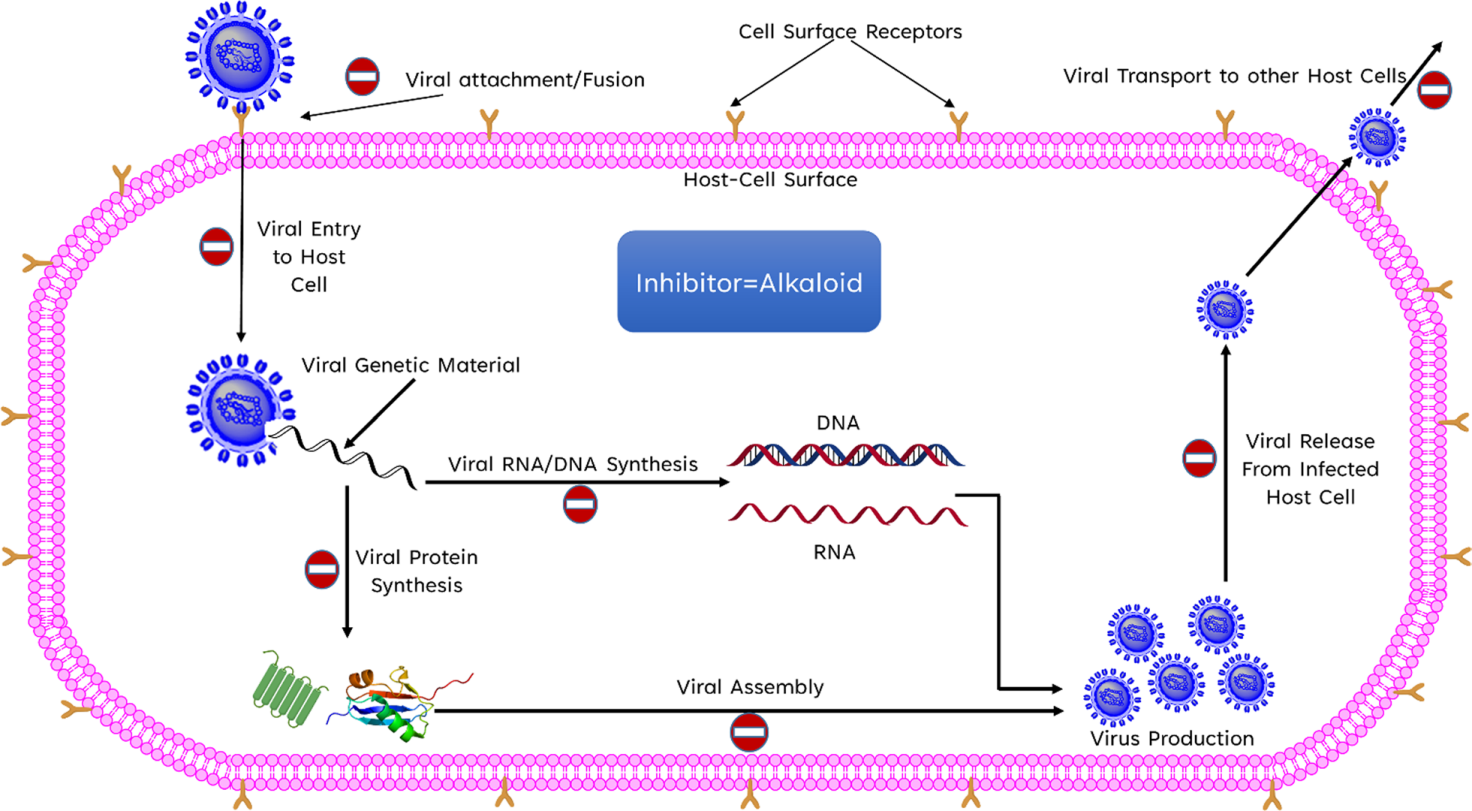
Alkaloids’ different modes of action against viruses (Adopted from Badshah et al. [141])

Figure 2 is a representation of the various modes of action of alkaloids against viruses discussed in this review paper. The antiviral action of these alkaloids includes targeting and inhibition of the transport of virus into the host, viral entry and fusion to the host cell surface, viral DNA and RNA synthesis processes, viral protein synthesis, and viral glycoprotein processing. All of the antiviral alkaloids discussed in this review and their sources (whether it is natural or synthetic) along with their chemical structures are given in Table 3.

**Table 3:** Antiviral alkaloids discussed here their structures and their sources

S.N	Alkaloid	Chemical structures	Source or origin	Ref.
1	Berberine		Natural	[19]
2	Homonojirimycin (HNJ)		Natural	[20]
3	Oxoglyantrypine		Natural	[21]
4	Norquinadoline A		Natural	[21]
5	Deoxynortryptoquivaline		Natural	[22]
6	Deoxytryptoquivaline		Natural	[22]
7	Tryptoquivaline		Natural	[22]
8	Compound-14		Synthetic	[23]
9	Yohimbine		Natural	[25]
10	Vincamine		Natural	[25]
11	Atropine		Natural	[25]
12	Trigonelline		Natural	[25]
13	Allantoin		Natural	[25]
14	Octopamine		Natural	[25]
15	Synephrine		Natural	[25]
16	Colchicine		Natural	[25]
17	Manzamine-A		Natural	[26]
18	Tetrandine		Natural	[27]
19	6-*O*-Butanoyl castanospermine		Synthetic	[28]
20	Tubingensin A		Natural	[31]
21	Cherylline		Natural	[37]
22	Castanospermine		Natural	[39]
23	Tomatidine		Natural	[40]
24	3-Oxo-voacangine		Natural	[41]
25	Voacangine-7-hydroxyindolenine		Natural	[41]
26	Rupicoline		Natural	[41]
27	Voacangine		Natural	[41]
28	Harringtonine		Natural	[16]
29	Piperine		Natural	[48]
30	Berberine chloride		Synthetic	[50]
31	Lycorine		Natural	[37]
32	Anisomycin		Natural	[38]
33	Emetine		Natural	[38]
34	Fusaindoterpene-B		Natural	[38]
35	JBIR-03		Natural	[58]
36	1,2-bis(1*H*-indol-3-yl) ethane-1,2- dione		Natural	[58]
37	Cephaeline		Natural	[47]
38	Ajmalicine		Natural	[66]
39	Chloroquine		Synthetic	[67]
40	Hydroxy-chloroquine		Synthetic	[67]
41	Pyronaridine		Synthetic	[67]
42	Homorringtonine		Natural	[72]
43	5,6-Dihydro-6-methoxynitidine		Synthetic	[75]
44	5-Methoxydictamine		Synthetic	[75]
45	Skimmianine		Natural	[75]
46	Sophaline B		Natural	[76]
47	Sophaline D		Natural	[76]
48	Sophaline F		Natural	[77]
49	Flavesine-J		Natural	[78]
50	Alopecurine-B		Natural	[78]
51	Dragmacidin-D		Natural	[81]
52	Fangchinoline		Natural	[82]
53	SIP-1		Natural	[83]
54	Manadomanzamine A		Natural	[86]
55	Manadomanzamine B		Natural	[86]
56	Xestomanzamine-A		Natural	[86]
57	Neokauluamine		Natural	[88, 89]
58	8-Hydroxymanzamine-A		Natural	[88, 89]
59	6-Deoxymanzamine-X		Natural	[88, 89]
60	12,28-oxamanamine-A		Natural	[88, 89]
61	Tulongicin-A		Natural	[90]
62	Dihydrospongotine-C		Natural	[90]
63	Michellamine D		Natural	[91]
64	Triptonine B		Natural	[92]
65	Michellamine F		Natural	[91]
66	Cepharanthine		Natural	[94]
67	Cyclopamine		Natural	[97]
68	A3E		Synthetic	[97]
69	Deoxynojirimycin		Natural	[101]
70	Jubanine G		Natural	[105]
71	Jubanine H		Natural	[105]
72	Nummularine B		Natural	[105]
73	*n*-Butyl deoxynojirimycin		Synthetic	[110]
74	(−)-12β-Hydroxyoxysophocarpine		Natural	[113]
75	(+)-12α-Hydroxysophocarpine		Natural	[113]
76	Aranotin		Natural	[114]
77	Gliotoxin		Natural	[115]
78	Homoharingtonin		Natural	[30]
79	Glaucine fumarate		Natural	[129]
80	*N*-Methyllaurotetanine		Natural	[129]
81	Isoboldine-HCl		Natural	[129]
82	Palmatine		Natural	[137]
83	Pericalline		Natural	[130]
84	Perivine		Natural	[130]
85	Periformyline		Natural	[130]
86	Leurocristine		Natural	[130]
87	Schizanthine Z		Natural	[70]
88	Thalimonine		Natural	[71]
89	Mefloquine		Synthetic	[67]
90	Capsaicin		Natural	[25]

## Conclusions

The alkaloid compounds and their synthetic analogues reviewed here exhibit robust antiviral activities against a wide range of infectious and deadly DNA/RNA viruses. Some of these compounds have IC_50_ concentrations lower than 5 µM and may prevent viral infections by more than 90 percent. Some alkaloids have higher antiviral activities than some standard antiviral medications, at lower concentrations. These reviewed alkaloids and their synthetic analogues were safe and nontoxic based on their cytotoxic concentration values ​​and other assays that we reported here. Whereas some compounds target a single stage of viral infection, others are multi-stage inhibitors able to strongly impede viral infection. The in vivo tests affirm that these compounds can protect mice from exposure to an otherwise fatal viral dose or protect them from viral diseases. Alkaloid compounds that have not yet been evaluated in vivo could be investigated further to determine their efficaciousness against these viral infections. We also found that the use of these alkaloids with other antiviral drugs in combination therapy can synergistically improve the efficacy of these standard medications and enhance antiviral activity to cure infections. Given the efficacy of these antiviral alkaloids in different experimental models, it is reasonable to conclude that these compounds have a high potential for development as antiviral medications against these infectious viruses. However, before these alkaloids and their derivatives can be used as antivirals, a better understanding of their pharmacological properties and clinical outcomes is required.

## Data Availability

All of the data analyzed during this investigation are accurately cited one by one and are easily accessible by following their respective references.
